# Exploring Stakeholder Perceptions of Facilitators and Barriers to Using Needle Exchange Programs in Yunnan Province, China

**DOI:** 10.1371/journal.pone.0086873

**Published:** 2014-02-03

**Authors:** Morgan M. Philbin, Zhang FuJie

**Affiliations:** 1 HIV Center for Clinical & Behavioral Studies Columbia University and the NY State Psychiatric Institute, New York City, New York, United States of America; 2 Treatment and Care Division, National Center for AIDS/STD Control and Prevention, Chinese Center for Disease Control and Prevention, Beijing, China; Johns Hopkins School of Public Health, United States of America

## Abstract

Injection drug use is an ongoing urban health crisis in China and one of the largest drivers of the transmission of HIV/AIDS. Sentinel surveillance sites in Yunnan province show upwards of 20% of injection drug users (IDUs) are HIV positive. Though the Ministry of Health has scaled-up needle exchange programs (NEPs), they have not received official government recognition nor have they been extensively evaluated to explore factors influencing their acceptability and feasibility. Using in-depth qualitative interviews conducted from February to July 2008 with 35 participants consisting of IDUs and other key stakeholders, we explored facilitators and barriers to accessing needle exchange programs in Kunming, the capital of Yunnan province. Content analysis was conducted to identify themes including attitudes toward NEPs and harm reduction, barriers to access, and suggestions for improvement. Themes that emerged included fears of breached confidentiality and police interference at the exchange sites and tensions between the public health and law enforcement perspective. Low levels of NEP-related knowledge and awareness were uniformly reported among interviewees. Suggestions to facilitate an increase in NEP acceptance included raising awareness of harm reduction and HIV more generally, offering services such as psychological counseling, job training and behavioral therapy at NEPs, and increasing communication between police, government, and public health officials. High rates of HIV infection among injection drug users in China have prompted rapid scale up of NEPs. Additional adaptations are necessary, however, to increase needle exchange use among injection drug users. This study finds that an urgent need to raise awareness of NEPs among policy makers and IDUs and act upon identified steps for developing social-structural interventions to create enabling environments that facilitate increased access to NEPs among injection drug users in Kunming.

## Introduction

In 2010, China had 1.35 million registered drug users, though there are estimated to be a total of 5–8 million [1]–[4]. Approximately 70–80% of drug users are thought to inject—predominantly heroin—with nearly 50% of injectors reporting needle sharing [5]–[9]. China detected its first AIDS case in 1985, and in 1989 HIV was diagnosed in an injection drug user (IDU) living in southern Yunnan [10], [11]. Injection drug use fueled China's early HIV/AIDS epidemic, and by 2002 HIV infection had been reported among IDUs in all 31 provinces and autonomous regions [12]–[14]. As of 2004, Chinese IDUs had an average HIV prevalence of 5.4%–8.2% [15], and in 2010, 88,798 registered drug users were HIV positive [5]. However, prevalence rates vary greatly by region with some sentinel surveillance sites in Yunnan, Xinjiang, and Guangxi reporting infection rates upwards of 75–89% among IDUs [5], [13], [16].

Harm reduction is a strategy to reduce the negative health, social, and economic effects of drug use, (e.g. the spread of blood-borne diseases), without requiring abstinence from drugs [17], [18]. Harm reduction has been particularly successful in lowering HIV rates within IDU-driven epidemics in cases where implementation and scale-up is swift [19], [20], and can include needle exchange, methadone maintenance treatment (MMT), peer education, and condom promotion. Though innovative mechanisms to provide clean needles (e.g. mobile clinics, vending machines and safer injection sites) have proven efficacious in other countries [21], [22], options in China are limited to the provision of sterile needles/syringes through exchange sites and peer educators [23]. In its expanded form, needle exchange programs (NEPs) can become a means of providing integrated health care, educational services, and referrals to drug treatment and HIV testing [24], [25]. NEPs have been evaluated within international settings and China itself, and found effective in preventing the acquisition of HIV and other blood-borne pathogens without increasing levels of drug use, discarded syringes, or crime [20], [26]–[29]. China began its first NEP in 2000 as a trial effort between local governments and international donors, and found it to be remarkably successful in lowering needle sharing and rates of Hepatitis C transmission [30], [31].

Though NEP and MMT are both considered pillars of harm reduction strategies, in China NEPs have not received the same amount of government support as methadone. Though national NEPs have been scaled up since 2004 [32] and are mentioned in China's second 2006–2010 action plan [30], there is no straightforward policy within Chinese written law and the ministry of public security has never officially sanctioned NEPs [1], [30]. These tensions highlight a potential conflict between the concept of harm reduction and the ways in which it is implemented and sustained. From a health perspective, NEPs are used to discourage needle sharing and lower the transmission of blood-borne diseases. From a security perspective, drug use is criminal and should be eliminated, and IDUs should be detained. As a result, NEPs are viewed as condoning drug use from a security perspective, but seen as beneficial in public health terms [20].

Within China, NEPs are only partially supported by government funding—international organizations provided 43% of funding for NEPs in 2004—in comparison to methadone clinics which are fully subsidized by the Chinese government [32]. This lack of formal support complicates implementation and compromises program success [20], [30], [31]. By 2008 there were 729 NEP sites located throughout China [30], [32] that were estimated to be reaching approximately 2% of all IDUs in the country [26], [33], or close to 40,000 IDUs [34]. Previous studies in China have attributed these low NEP attendance rates to lack of awareness of the service, limited choice in the types of needles provided, inconvenient hours and locations, and fear of police arrest [32], [35], [36]. The high HIV prevalence in this population, coupled with the low percentage of IDUs using NEPs, highlights the importance of addressing injection drug use and incorporating harm reduction measures into public health policy within China.

In this paper we examine perceptions and attitudes among Chinese IDUs and other key stakeholders toward harm reduction services as well as the facilitators and challenges to accessing NEPs in particular. Key stakeholders include peer educators (often former drug users serving as outreach workers), MMT clinic staff, and program staff of organizations working on HIV and drug use. This study aims to fill the gap of qualitative research knowledge to inform ways in which public health professionals develop culturally appropriate interventions to increase access and retention at NEPs in China.

## Setting and Methods

The research was conducted in Kunming City, Yunnan Province, a city of 3 million people in southwestern China [37]. Yunnan Province borders Burma, Laos, and Vietnam, which make up the ‘Golden Triangle’, one of Asia's main opiate producing regions. As of October 2007, 40,000–50,000 people in Yunnan were HIV-positive [5], 65% of whom were infected through sharing non-sterile syringes [38].

From February-July 2008, 20 IDUs were recruited through NEPs, a drug rehabilitation center, and methadone clinics, and were deemed eligible to participate if they were at least 18 years old and had self-reported drug use in the last year. Additionally, 15 key stakeholders were recruited from local and international non-governmental organizations (NGOs) working on issues of drug use, HIV/AIDS, or harm reduction, and included individuals working as peer educators, program coordinators, and drug rehabilitation and methadone clinic staff.

Purposive sampling was used to recruit individuals who had experience with injection drug use, needle exchange programs, and harm reduction more generally, and could thus provide insight into knowledge and barriers surrounding access to harm reduction programs. In this instance it was used to recruited current and former injection drug users who could speak to accessing and using needle exchange programs, as well as NGO-staff and policy makers who could address to the broader socio-political context.

### Data Collection and Analysis

Qualitative data collection methods were used to elicit detailed narratives about participants' experience with drug use and harm reduction without imposing pre-existing assumptions [39]–[41]. Written informed consent was obtained from each injection drug user, and oral consent was obtained from each stakeholder. Oral consent consisted of reading the consent document aloud to the participant, asking if they had any questions, and then asking if they were still willing to proceed with the study. If they agreed, the researcher initialed and dated the oral consent form. This study was approved by Institutional Review Board at the Chinese Centers for Disease Control and Prevention. The IRB approved verbal consent for stakeholders since these stakeholders were speaking about their professional experiences in the fields of HIV and drug prevention and drug policy (as opposed to the drug users who were speaking about personal experiences). Interviews were semi-structured yet allowed for additional probes when salient topics arose. The interview guide focused on the following topics: the role of drug use in Chinese history, how an individual began using drugs or first became involved in the field of drug prevention, perceptions of harm reduction and NEPs, facilitators and barriers to access and use, experience (if any) with detention, drug cessation, NEPs, or methadone, understanding of local and national-level drug policies, and suggestions for further improvements. Specific questions included: 1) Please tell me about the first time you used drugs; 2) Have you ever tried to stop using drugs? Can you tell about what you did; 3) What have you heard about needle exchange programs? Please tell me about any personal experience you have with these; and 4) Have you ever had any interactions with police as a result of your drug use? If so, please describe to me what happened. The first author conducted interviews in private locations including rehabilitation centers, NGO offices, and places of work. The IDU participants were compensated 25 Chinese Yuan (∼$4) for their time.

Interviews were conducted in Mandarin Chinese, digitally recorded, and ranged from 35–75 minutes (average 45 min). Native Chinese speakers conducted verbatim transcription and translation, with translations validated by bilingual individuals. Digital files were destroyed after transcription. A “do not translate” list including street jargon and slang was created to preserve the meaning of key concepts in the original Chinese.

Thematic analysis focused on accessing and using NEPs in the Chinese context, structural facilitators and barriers, and suggestions for future improvement. Transcripts were first hand-coded by the authors who, after reading a subset of transcripts, created a preliminary codebook containing key categories. These codes were applied to ten interviews to ensure applicability and allow for the creation of more nuanced versions of the codes. The remaining interviews were hand coded then uploaded into ATLAS.ti [42] and electronically coded. Any discrepancies were discussed among investigators and resolved.

## Results

The mean age for IDUs was 35 years (range 26–43) and 36 years for NGO staff (range 29–55). Of the IDUs interviewed 55% had completed high school whereas among NGO staff participants the rate of high school completion was 67% (among whom 40% completed college or higher). Of the 20 IDUs, approximately half reported ever attending a needle exchange program, as did 2 of 5 peer educators (who were previous injectors). Nearly all NGO staff reported daily interactions with IDUs, and half interacted regularly with a needle exchange program. Though IDUs and NGO staff differed widely in their education and professional history, their beliefs regarding challenges to harm reduction utilization in China were similar. As a result, data from IDUs and NGO staff were combined for analysis.

In data analysis, three general themes emerged: 1) general responses to drug use, harm reduction and needle exchange programs, 2) challenges to access and uptake, and 3) suggestions for improving existing services. These themes are described below.

### General Responses to Drug Use and Harm Reduction:

#### Drugs as a part of colonial history

The majority (85%) of individuals interviewed supported harm reduction because of its demonstrated ability to decrease levels of blood-borne infections, reduce crime, and help IDUs integrate into society. However, participants stressed the specificity of the Chinese context and how the broader cultural-historical framework must be taken into account when discussing drug use and harm reduction. More specifically, drug use was framed against a backdrop of the opium wars that China conducted with Britain in the 1800s. As one NGO worker described:

“The culture is a barrier to harm reduction due to China's relationship with drugs, especially heroin. This is a Confucian society and there is a historical barrier to drug use based on its association with the western powers, [that of] humiliation and control. The Korean and British kingdoms around China have for hundreds of years used drugs as a way to sedate China.” (NGO_Male_37)

#### Conflicting views on needle exchange programs

Many participants pointed out the importance of needle exchange programs in reducing infectious disease transmisison:

“In Kunming, there are many HIV-infected individuals due to the sharing of needles. When they exchange needles they want to save money on buying new needles; people don't like to spend that money. If you use mine [needle] and I use yours, it's not very healthy.” (NGO_Female_30)

In contrast, one IDU noted that although he would support methadone because it required people to stop drug use, which he felt would have a positive influence on law and order, he was wary of NEPs:

“Methadone is definitely more effective because it encourages everyone to quit using drugs. If drug abuse spreads it creates a problem for public security, which will lead to social turmoil. Providing needles alone in the context of needle exchange is a way to encourage drug using. It's like saying, you use drugs? No fear, come to us if you need more needles.” (IDU_Male_40)

The majority of participants expressed strong support for NEPs, citing that many IDUs were not ready or able to quit. Others pointed out that NEPs filled an important gap in locations where pharmacies are unwilling to sell needles to drug users, or when IDUs are unable to afford new needles. As one individual noted:

“Needle exchange is definitely successful. Based on my personal experience, and the experience of people around me, if you have money to buy drugs, there would be no money to buy needles, so you share, which could lead to serious consequences….So it solves both psychological and financial burdens.” (IDU_Male_40)

### Challenges to the use and scale up of NEPs

Though the majority of individuals supported harm reduction and noted the importance of NEPs, they discussed numerous challenges to their use and scale-up. The most salient barriers included lack of knowledge about NEPs among IDUs and fears regarding confidentiality and police interference.

#### Lack of awareness about NEPs

Even though participants in this study were recruited through NGOs, rehabilitation centers, and methadone clinics, a number of IDUs reported not knowing NEPs existed in their area or how they function.

“They don't know about it. The program is not offered in many areas, and many people have never heard of such things. They just don't know about it – it's not that they don't want to participate in it.” (IDU_Male_36)

In addition, many reported that their friends did not take blood-borne diseases seriously, and that the benefits of attending an NEP was outweighted by other risks they faced such as arrest or incarcertation:

“They [IDUs] distrust the government and wonder if they will get caught or not. If they were arrested for only a couple of needles it wouldn't be worth it…I think there is the problem of not taking it too seriously. Most do not realize this disease's severity, so for them, the topic is a boring topic. If they bought one needle and used it for 5 days, it's not as troublesome as buying a new one each time.” (IDU_Male_32)

In addition to increasing knowledge of NEPs among IDUs and increasing the number of NEP distribution sites, participants also said that NEPs should be managed in ways that make the services more accesible to IDUs. Maintaining NEPs in convenient locations with extensive hours was considered important because, as one NGO official noted, IDUs are only willing to travel so far to access services:

“My feeling is that [the government] is doing an incredible job of scaling up hardware more so then software and that they are outpacing all other places in the world. However, they build these centers but it doesn't mean people will come. We have it and it's not used. They need to be in safer zones, the times are a problem, and the location. If I'm a drug user I'm not going to get on a bus for one and half hours or pay for a taxi. If it closes at 5pm I can't help it.” (NGO_Male_37)

#### Concern with Confidentiality

A recurring theme described by IDUs and NGO workers was the fear that presenting at an NEP would serve to disclose an individual's status as a drug user, which could lead to discrimination, stigmatization, or arrest. Well over two-thirds of participants mentioned this fear. As one IDU explained:

“Some people are skeptical. They don't trust the government and other social organizations. They are afraid that others might find out that he is a drug addict. I haven't participated, because personally I am afraid that others will know that I am still abusing drugs.” (IDU_Male_36)

A peer educator noted that in order to ensure confidentiality, NEPs should be mobile and go to wherever IDUs are, whether that means meeting them in their house, a shooting gallery, or specified street-corner.

#### Police and Public Security

The most consistently reported barrier to accessing NEPs was interference of NEP operations by local police security authorities. NGO staff stressed the need for policy makers to create new laws to address these challenges, and injection drug users spoke of a need for education and training among police. Specific barriers include police harassment and fear of targeted arrests by police waiting for clients to leave NEPs. According to these comments, arrests are more common toward the end of the month. As one individual explained:

I think the main barrier is the public security bureau. No matter how good the relationship is between the Center for Disease Control and the Public Security Bureau, there will be tension. I have heard plenty of stories of the police hanging out outside of clinics and arresting people… So that is a huge problem. People are afraid to present at the harm reduction places when the cops are around, and it also limits outreach that can be done into the drug using community. (NGO_Male_53)

An IDU echoed this problem of police interference and how it deters people from presenting at harm reduction facilities, but also stressed how the government can work to eliminate this barrier.

“There are police watching and waiting to arrest people. It's embarrassing if you got caught while exchanging needles and most of my friends do not like to go because the police might find out. It's not worth it because on needle only costs 1 or 2 Yuan (∼12–25 cents). It would be best if the police didn't go interfering because they scare everyone off…The government gives the police too much pressure to arrest some number of people in a certain amount of time, they can't help but look everywhere to find the needle exchange programs. It doesn't matter if you're injecting or exchanging, they will arrest you as long as they see you.” (IDU_Male_45)

Though this IDU noted how the government might work to eliminate this barrier, those interviewed did not express faith that the government wished to eliminate these types of barriers. Both NGO-level staff and IDUs described police presence as a barrier that strongly influenced the ability to provide, and access, clean needles. Nearly all individuals we spoke with felt strongly that both national and local level policy must be better harmonized in order to provide effective services, and that what was occurring on the ground should match the stated policies. As one individual noted:

“It (NEP) is a very successful intervention. But it's a very contradictory subject because on one hand, the government has put in a lot of funding to maintain this program and prevent HIV transmission, but on the other hand, the police department is attacking it. Isn't it ironic.” (NGO_Female_38)

### Suggestions for improving access to NEPs

Participants raised various suggestions for improvement of NEPs, including facilitating discussions between the national government and security bureau, increasing awareness of NEPs among both IDUs and the general community, and providing a broader array of social services for IDUs. Many cited the discrepancy between the positions of the ministry of health and the police, noting the need for closer cooperation between the two. As one individual explained:

“There is a very different view in China than in the U.S. For example we think of harm reduction as being there to lower levels of HIV/AIDS, but here, it is more a question of public order and control as opposed to health. They want to get people off drugs and are not so worried about whether they might get infected.” (NGO_Male_52)

This individual's sentiments suggests that, on some level, China is more focused on maintaining control of the flow of drugs, and those who choose to use them, than the potential public health ramifications. This belief may stem from a disconnect in the rapid scale-up of methadone clinics in China in comparison to a relative lack of support for NEPs; it may also reflect the power the public security bureau has in policy formation and implementation as opposed to the CDC and public health focused organizations. This tension between public security and public health was consistent throughout the interviews.

Many of the NGO staff felt strongly about the need to link drug users accessing one type of harm reduction treatment to other harm reduction alternatives (e.g. needle exchange programs to methadone), in order to move them along a path toward cessation. This included working directly with the rehabilitation centers to inform drug users of available community services incase of relapse, and linking methadone and NEPs so IDUs knew of available options when they were ready to stop using drugs. As one individual in the NGO sector described:

“First we do the needle exchange, and then I will tell them about methadone to quit drugs. I will tell them to quit and drink methadone every time they are here, but they are sometimes unwilling to listen. We have to be patient and explain to them one step at a time, then they will listen.” (NGO_Female_43)

Some interviewees felt that in addition to needle exchange and methadone maintenance, there was an unmet need for psycho-social support and job training for those seeking work following recovery from addiction.

“There should be a psychiatrist to go to when you are feeling depressed or sad while using drugs. There should be someone who understands you…to help us to find a job and to seek emotional help. We are already isolated from society, so it would be most beneficial if they could provide us employment opportunities.” (IDU_Male_45).

## Discussion

Based on our knowledge, this is the first qualitative study to examine attitudes and barriers towards needle exchange programs in Kunming, China. Interviews with current and former drug users and NGO workers can better inform our understanding of how these groups perceive barriers such as lack of knowledge among IDUs, police, and policy makers, confidentiality concerns, and police interference, as well as ways in which they feel such programs would be improved.

Reports of stigma and discrimination are common among drug users, and though media campaigns have been conducted to improve awareness surrounding HIV/AIDS and drug use, knowledge and acceptance of NEPs remain low among community members [30]. Though acceptance of NEPs is high among IDUs, and many report wanting to access them, there is a fear that it may result in incarceration. Both NGO-staff and IDU respondents suggested raising community and IDU awareness, and in turn hopefully lowering stigma through media and school-based campaigns, collaboration with community leaders, and families of IDUs. Studies have found that IDUs who are more aware of the benefits of harm reduction are more likely to participate [18], [43], [44]. Numerous NGO participants also suggested providing information about harm reduction services to drug users in detoxification centers since relapse rates are close to 95% and IDUs are often unaware of harm reduction services, a strategy widely supported by studies of post-release relapse and recidivism. [20], [45], [46]. The provision of information within detention centers could include having local NGOs offer information about their services in case detainees need additional post-release assistance, with a focus on support groups, NEPs and methadone programs.

Discrimination of IDUs remains high in China, and many IDUs expressed reluctance to attend harm reduction centers due to the lack of confidentiality and fear that others would discover their status as a drug user [18], [43], [47], [48]. Some programs have tried to address issues of confidentiality by having mobile vans serve as NEPs, or even NGO-members who carry clean needles in their backpacks and go directly to the houses of drug users to exchange needles [21], [22], [49]. Others also feared arrest and harassment by police who might identify them through their attendance at harm reduction services. A Burnet Institute Report noted that 10–30% of IDUs spend time in a mandatory detoxification center each year [50], and studies in the United States have reported similar barriers to harm reduction: punitive eligibility requirements, fear that attendance will reveal one's IDU status, and fear that NEPs may lead to increased loitering and the creation of heroin markets near clinics [51], [52].

Many who were interviewed felt that an advantage of NEPs was their appeal to current users, which could be an important tool for delivering other harm reduction services to this hard to reach population. Some suggested integrating NEPs and methadone clinics at the same location so center staff could build rapport with IDUs while exchanging needles and socialize them to the concept of methadone maintenance through staff they know and trust. Previous studies in China have found that IDUs perceive NEPs to be less risky than methadone because NEPs allow them to participate from within the community and without registration [31], [53]. This situation makes NEPs particularly effective for initiating contact with IDUs without an arrest record, and it can also facilitate accessing marginalized IDU groups such as minorities and migrants [20], [31]. Greater links between NEPs and MMT programs can function as two-way referrals for IDUs seeking methadone treatment as well as those patients who drop out of methadone programs [31], [54].

Participants also emphasized the importance of additional services including job training skills, housing support, and psychological counseling. Psychological support and therapy is important for both its emotional benefits and its ability to increase effectiveness and retention rates within harm reduction centers [48], [55], [56]. Though the social work sector in China is still relatively underdeveloped many respondents felt strongly that they would benefit from such services [19], [43].

The most commonly discussed problem among respondents related to law enforcement and fear of arrest [57]. Law enforcement as a barrier to accessing harm reduction services has been reported in numerous settings including Russia, China, Canada, Vietnam, and the United States [13], [32], [47], [58]–[60]. An evaluation of fifteen NEPs in China found that police attitudes affected rates of needle turnover, and that NEP staff and police must work closely to facilitate IDUs' use of NEPs [32]. Though health officials endorse harm reduction, support for harm reduction is limited at high levels of the government and among public security authorities who see it as condoning drug use [26],[32]. This has translated into police practices on the ground in which officers target drug users not only for reasons of public security but also in response to pressure from superiors to meet arrest quotas [26],[37],[53]. Many studies have shown police support for total abstinence (as opposed to harm reduction), as a result, one approach to working with the public security bureau could be to emphasize that participation in a syringe access intervention often facilitates cessation [24],[25],[54],[61]. Another approach might be facilitating national-level dialogue between the public service bureau and Centers for Disease Prevention and Control to assess how best to address both public health and policing needs.

The aforementioned barriers highlight an ongoing tension between advocates of harm reduction, who see it as a means of lowering blood borne illnesses, and those who see it from a security perspective and as something that should be handled through criminalization [20]. Though the central government has pushed for increased cooperation between the involved ministries—public security, justice, education, health—implementation varies greatly at the local level [20],[30],[62], highlighting the need for support at each level of government. Support from high-level leaders is especially important in China due to its highly centralized policy development process and decentralized implementation process [31]. A lack of consensus among public security leaders, however, has resulted in ambiguous statements regarding harm reduction, which has contributed to confusion at a local level regarding how officials should implement policy [53]. Studies have also suggested working with the Chinese government—specifically local and provincial leaders—to increase understanding of drug use and focus on a more treatment-oriented approach rather than punishment [13],[26],[63].

One limitation of this study is that some interviewees did not have extensive knowledge of harm reduction interventions. Also, although we conducted interviews across various organizations, and varied between those who interacted daily with IDUs and higher-level coordinators, we were unable to interview any police or detention facility personnel. We worked with the NGO in Kunming to reach out to such officials but none were willing to participate. Although this study was only conducted in one city in southwestern China, the results align with existing studies from other countries, suggesting its salience. Due to the exploratory nature of the study and, we focused on applying a thematic analysis to understand emergent concepts as opposed to grounding the research in a already existing conceptual framework. Though we did not use a theoretical sampling framework, certain themes were repeatedly mentioned across participants, suggesting that the data had reached saturation and the results were meaningful. In addition, these themes were generated through both IDU and other stakeholder perspectives, which enhances credibility.

Findings from this study suggest that while the Chinese Ministry of Health's support of harm reduction has been a positive step forward, additional work is required to scale-up NEPs and encourage attendance among IDUs. Respondents believe it is crucial to address an underlying wariness toward NEPs, and to have the policy makers, public security bureau, and NGOs increase their levels of coordination to help facilitate the privacy of IDUs attending these clinics, and lower their fears of arrest. Facilitating dialogue and collaboration between the ministry of health and public security bureau and increasing awareness may move critical interventions forward. In the meantime, existing NEPs throughout China will continue to provide services to IDUs in the effort to lower rates of drug use and help stem China's HIV epidemic.
